# Hydrogen Evolution Reaction on Ultra-Smooth Sputtered Nanocrystalline Ni Thin Films in Alkaline Media—From Intrinsic Activity to the Effects of Surface Oxidation

**DOI:** 10.3390/nano13142085

**Published:** 2023-07-17

**Authors:** Daniela Neumüller, Lidija D. Rafailović, Aleksandar Z. Jovanović, Natalia V. Skorodumova, Igor A. Pašti, Alice Lassnig, Thomas Griesser, Christoph Gammer, Jürgen Eckert

**Affiliations:** 1 Department of Materials Science, Montanuniversität Leoben, 8700 Leoben, Austria; daniela.neumueller@unileoben.ac.at (D.N.);; 2 University of Belgrade-Faculty of Physical Chemistry, 11158 Belgrade, Serbiaigor@ffh.bg.ac.rs (I.A.P.); 3 Department of Materials Science and Engineering, School of Industrial Engineering and Management, KTH–Royal Institute of Technology, 100 44 Stockholm, Sweden; snv123@kth.se; 4 Applied Physics, Division of Materials Science, Department of Engineering Sciences and Mathematics, Luleå University of Technology, 971 87 Luleå, Sweden; 5 Erich Schmid Institute of Materials Science, Austrian Academy of Sciences, 8700 Leoben, Austria; 6 Institute of Chemistry of Polymeric Materials, Department of Polymer Engineering and Science, Montanuniversität Leoben, 8700 Leoben, Austria

**Keywords:** hydrogen evolution reaction, magnetron sputter deposition, electrochemical surface oxidation, electrolysis, water splitting

## Abstract

Highly effective yet affordable non-noble metal catalysts are a key component for advances in hydrogen generation via electrolysis. The synthesis of catalytic heterostructures containing established Ni in combination with surface NiO, Ni(OH)_2_, and NiOOH domains gives rise to a synergistic effect between the surface components and is highly beneficial for water splitting and the hydrogen evolution reaction (HER). Herein, the intrinsic catalytic activity of pure Ni and the effect of partial electrochemical oxidation of ultra-smooth magnetron sputter-deposited Ni surfaces are analyzed by combining electrochemical measurements with transmission electron microscopy, selected area electron diffraction, X-ray photoelectron spectroscopy, and atomic force microscopy. The experimental investigations are supplemented by Density Functional Theory and Kinetic Monte Carlo simulations. Kinetic parameters for the HER are evaluated while surface roughening is carefully monitored during different Ni film treatment and operation stages. Surface oxidation results in the dominant formation of Ni(OH)_2_, practically negligible surface roughening, and 3–5 times increased HER exchange current densities. Higher levels of surface roughening are observed during prolonged cycling to deep negative potentials, while surface oxidation slows down the HER activity losses compared to as-deposited films. Thus, surface oxidation increases the intrinsic HER activity of nickel and is also a viable strategy to improve catalyst durability.

## 1. Introduction

One main aim and indispensable development in our society is decarbonizing energy considering the related climate change. Technological progress already allows the generation of sustainable green energy by wind turbines, photovoltaic plants, and solar power for competitive prices. A challenge, however, is the temporal and local dependency of renewable energy sources. Thus, suitable energy storage is needed to efficiently provide green energy independent of its natural fluctuations [1]. Therefore, hydrogen is considered to play a key role in the energy transition, serving as a green energy vector. However, worldwide hydrogen production relies almost entirely on fossil resources, while sustainably-produced hydrogen through water electrolysis still faces high production costs and limited availability [2]. To compete with well-established fossil energy carriers, the generation of green hydrogen needs to become more effective in terms of energy conversion and connected prices.

One crucial field of improvement is the research of electrocatalytic materials to boost electrolytic water splitting. Due to the sluggish reaction kinetics of both half-cell reactions, decreasing the overpotential of the hydrogen evolution reaction (HER) and the oxygen evolution reaction (OER) is one crucial parameter in lowering the energy barrier and, therefore, decreasing the overall process energy consumption [3,4].

The efficiency of the cathodic HER in alkaline media is strongly connected to the adsorption and desorption behavior of reaction educts, products, and intermediates. The optimum catalytic efficiency is reached with intermediate binding energies. Weak binding of intermediates leads to inefficient reaction activation, while strongly bound intermediates might block the surface and could poison the catalyst [5,6]. For the HER, the free energy of hydrogen adsorption is an indicator of catalyst activity [5] and could be applied as a descriptor in different electrolytes [7].

The HER in the neutral and alkaline electrolytes is described by the following steps [8]:

H_2_O + e^−^ + * → H_ads_ + OH^−^ (Volmer step) (1)

H_ads_ + H_2_O + e^−^ → H_2_ + OH^−^ + * (Heyrovsky step) (2)

2H_ads_ → H_2_ + 2* (Tafel step) (3)

where (*) stands for the adsorption site on the catalyst surface. The cathodic reaction proceeds either via the Volmer–Tafel ((1) and (3)) or via the Volmer–Heyrovsky route ((1) and (2)). The Volmer step (1) describes the dissociation of a water molecule and the formation of an adsorbed hydrogen species, H_ads_. Additionally, during electrolysis, the charge carrier OH^−^ is formed. The role of OH^−^ adsorption on the catalyst surface is a matter of recent intense studies in determining catalytic activity [9,10,11]. In the Tafel step (3), H_ads_ can recombine with another H_ads_, forming an H_2_ molecule which ideally would leave the surface in the form of gas. Another option for the second reaction step is the Heyrovsky reaction, where H_ads_ combines with a water molecule, generating a hydroxide anion in addition to the hydrogen molecule (2).

In contrast to acidic water splitting, alkaline electrolysis suffers from more sluggish reaction kinetics due to an additional water dissociation step, creating an extra energy barrier [12]. Not only is the catalyst–hydrogen (M-H) binding energy shown to be relevant, but water and hydroxyl ion adsorption energies are also of major interest [11,13]. Furthermore, factors like the electrochemical double layer and the potential of zero free charge can influence the overall kinetics [14]. 

To lower the arising energy barriers, three main approaches can be emphasized in developing novel catalytic materials, including manipulating the electronic structure, the design of specific intermediate adsorption sites, and adjusting the catalyst surface [9]. Factors like improved conductivity and an enlarged active surface area can effectively improve the catalytic efficiency [14]. Further, tailoring supported catalysts acknowledging the active role of the support material is a feasible method [15].

Concerning price reduction, the industry is interested in producing non-noble metal, high surface area structures to limit the use of material and maximize efficiency in upscale applications. However, limited work has been done on fundamental aspects of thin layers and flat surfaces, although this is relevant for benchmarking the material’s intrinsic catalytic activity and determining layer thickness influences which ultimately provide essential data for necessary material consumption. Therefore, this work aims to study the intrinsic material properties of an abundant element as Ni, rather than roughness and surface area effects. The latter imposes significant problems in evaluating the intrinsic catalytic activity of Ni surfaces as there is no reliable electrochemical method for determining the electrochemically active Ni surface area.

Concerning availability and economic factors, industrial alkaline electrolysis uses either stainless steel or Ni catalysts, as they present the best non-noble alternatives to expensive, platinum group metal catalysts [16,17,18]. Due to its good activity and stability in alkaline solutions, a fundamental understanding of the HER mechanism on Ni surfaces is a relevant topic of current research from both fundamental and application perspectives. It has already been shown that the presence of transition metal oxide species influences the catalytic behavior of pure metal catalysts like Pt and Ni [19,20,21]. Metal|metal oxide heterostructures were shown to possibly have a positive influence on the shift of HER overpotentials to energetically more favorable ones [22]. In the case of Ni, the presence of oxide species on the surface is considered a possible factor in tailoring surface activities and creating potential reduction sites. The interplay of Ni and its oxide species (NiO, α-/β Ni(OH)_2_, NiOOH) has been extensively investigated before, showing that nickel oxide species positively influence the activity and reaction overpotential via tailoring the adsorption behavior of intermediate species [21,23,24].

However, bulk NiO and Ni(OH)_2_ show poor electrocatalytic behavior, mainly attributed to the unfavorably high hydrogen adsorption energy [23]. Therefore, a synergistic effect between Ni and NiO/Ni(OH)_2_ induces superior catalytic activities. For this effect, surface Ni^0^ provides optimized conditions for hydrogen adsorption, while the formation of OH_ads_ should be promoted by surface oxide species (NiO) [11,25]. It should also be highlighted that the release of reaction products is facilitated by oxide species on the surface, thus preventing the occupation of the surface and its blocking [24]. However, a reliable estimation of real activity increases by the formation of NiO|Ni interfaces also suffers from the abovementioned obstacles related to real (electrochemically active) surface area measurements. 

This work concentrates on the preparation and evaluation of the HER activity of ultra-smooth polycrystalline Ni thin films and the effect of electrochemical oxidation on the catalytic activity of its modified surfaces towards HER in alkaline media. In this work, we aim to evaluate the intrinsic catalytic properties of Ni and the effect of in-situ electrochemical oxidation on the catalytic behavior simultaneously, while precisely monitoring the evolution of the surface state and the surface roughness of the deposited Ni films. The present experimental work is supplemented by Density Functional Theory (DFT) calculations and Kinetic Monte Carlo (KMC) simulations to obtain a deep atomic-level understanding of the HER on oxide-modified Ni surfaces and the role of the rates of different elementary processes involved in the HER mechanism. Finally, we analyze the impact of prolonged Ni film cycling to deep cathodic potentials while monitoring the evolution of surface topology and local crystal structure. 

## 2. Materials and Methods

### 2.1. Preparation of Ni Thin Layers

Low roughness Ni thin film electrodes were produced by DC magnetron sputtering. The metal layer was deposited on various substrates, specifically on Cu substrate with enhanced adhesion, as well as on silicon wafers for layer thickness evaluation. Before deposition, the Cu foil was mechanically cleaned and polished to minimize the original roughness. Subsequently, the substrate was washed thoroughly with deionized water and isopropanol. The substrates were etched in H_2_SO_4_ (*v*/*v* 10%) for enhanced adhesion and rinsed with isopropanol. The cleaned substrates were used immediately to prevent the formation of an oxidation layer, which negatively influences the adhesion of the sputter-deposited layers.

Magnetron sputter deposition was performed with a HEX series Compact Thin Film Deposition System by Korvus Technology equipped with a Fission DC Sputtering Source. Due to the magnetic properties of Ni, the strong magnetron available from Korvus Technology was required for deposition. The Ni sputtering target with a purity of 99.9%, a diameter of 50.8 mm, and a thickness of 1 mm was obtained from HMW Hauner GmbH & Co. KG (Röttenbach, Germany). The targets were pre-sputtered for three minutes before every deposition. The deposition power was chosen to be 80 W with an argon flow of 20 sccm and a total constant pressure of 1.4 × 10^−3^ bar. The sample stage rotation was set to 20 rpm for homogenous deposition on the substrates with a fixed sample stage to target distance of 12 cm. The deposition time was selected to produce Ni samples of about 50 and 300 nm thickness, yielding actual layer thicknesses of 46 ± 8 nm and 287 ± 4 nm. For simplification, the layers will be referred to as 50 and 300 nm films, respectively.

### 2.2. Materials Characterization

Layer thickness, deposition rates, and estimation of the roughness of the electrodes were obtained by laser scanning confocal microscopy with an Olympus LEXT OLS4000 3D laser microscope (Tokyo, Japan). Determination of the thickness was performed for Ni layers on Si wafers, enabling better contrast in the specimens.

Transmission electron microscopy (TEM) imaging was done on a Philips CM 12 TEM with an acceleration voltage of 120 kV. The TEM samples were directly sputter deposited on electropolished Cu support.

Topographic images of the thin films were recorded with a Bruker Dimension Icon atomic force microscope (AFM). The AFM was operated in intermittent contact mode with a TESPA-V2 etched silicon probe (Bruker) with a typical spring constant of 37 N m^−1^, a resonance frequency of 320 kHz, and a tip radius of 7 nm. Image processing and evaluation were executed using Gwyddion 2.61 [26] data analysis software. All presented images were processed using step-line correction. Before any roughness evaluation, images underwent flattening to eliminate curvature originating from sample mounting. 

X-ray photoelectron spectroscopy was conducted with a Thermo Fisher Scientific Inc. Nexsa G2 photoelectron spectrometer system equipped with a low-power Al K-Alpha X-ray source yielding a 30–400 µm adjustable X-ray spot size. Survey spectra were recorded for binding energies up to 1350 eV for each sample. Additionally, Ni 2p (840–890 eV) spectra and O 1s (525–545 eV) spectra were recorded.

### 2.3. Electrochemical Measurements

Electrochemical measurements were conducted with a PARSTAT4000A potentiostat using VersaStudio 2.63.3 software and a three-electrode setup. The working electrodes were the produced sputter-deposited Ni thin films on the Cu substrate. The electrodes were contacted and electrically insulated to avoid Cu contribution during the analysis. A Pt coil served as the counter electrode. We note that Pt counter electrode dissolution followed by Pt re-deposition on the working electrode was not observed using XPS. All measurements were conducted with an ALS Co., Ltd. (Tokyo Japan) Hg/HgO reference electrode for alkaline solutions and were performed in 1 M KOH at room temperature. The employed electrolyte was prepared from KOH pellets (99.99% purity; Sigma-Aldrich, Darmstadt, Germany) dissolved in deionized water. The iR drop was compensated in all cyclic voltammetry (CV) measurements up to 75% of the electrolyte resistance, which was determined via single-point impedance measurements (E = −0.8 V vs. Hg/HgO, 100,000 Hz).

To analyze the catalytic activity towards the HER and monitor the effect of electrochemical oxidation of the samples, the working electrode was first used as a cathode examining the HER by cyclic voltammetry in the region from −0.6 V to −1.3 V vs. Hg/HgO. For electrochemical oxidation of the films, the samples were cycled in the anodic regime from −0.4 V to 0.7 V vs. Hg/HgO. The electrode was cycled from −0.6 V to −1.3 V vs. Hg/HgO after the electrochemical oxidation process to monitor the influence of oxidation on the catalyst activity. A sweep rate of 5 mV s^−1^ was chosen for all cyclic voltammetry experiments. For further comparison of acquired data and calculations, the back loop of the third cycle was selected. For reproducibility, all experiments were performed at least three times. Comparative data were collected from the back-cycling curve at −10 mA cm^−2^. As benchmark catalysts, electrodeposited Ni and Pt layers from McCrory et al. were chosen [27].

To test the durability of the sputter-deposited Ni thin films during HER, cyclic voltammetry in the range of −0.6 V to −1.3 V vs. Hg/HgO and a sweep rate of 5 mV s^−1^ was performed. In total, 100 cycles were recorded to monitor the evolution of the catalyst activity and analyze the influence of the surface oxide. The cell was purged with N_2_ continuously during the measurements.

The potentials provided in this work were converted from Hg/HgO to the Reversible Hydrogen Electrode (RHE) by the following equation:

E_RHE_ = E_Hg/HgO_ + 0.098 V + 0.059 V × pH (4)

### 2.4. Computational Modeling

First-principles Density Functional Theory (DFT) calculations were performed using the Vienna ab initio simulation code (VASP) [28,29,30]. The Generalized Gradient Approximation (GGA) in the parametrization by Perdew, Burk, and Ernzerhof [31] combined with the projector augmented wave (PAW) method was used [32]. Cut-off energy of 600 eV and Gaussian smearing with a width of σ = 0.025 eV for the occupation of the electronic levels were used. Spin-polarization was included in all the calculations. The first irreducible Brillouin zone was obtained using a Γ-centered 4 × 4 × 1 grid utilizing the general Monkhorst–Pack scheme [33].

The Ni(111) surface was modeled using a (4 × 4) supercell with 5 Ni layers. During structural relaxation, two bottom layers were kept fixed in their bulk positions, while other layers were allowed to fully relax until the forces acting on the atoms dropped below 0.01 eV Å^−1^. To investigate the interface between metallic nickel and oxidized nickel phase as catalytically active sites, a (NiO)_2_ fragment was placed on the surface of the Ni(111) slab. To quantify the interactions between adsorbates (OH and H) with (modified) Ni(111) surface, the binding energies (E_b_) were calculated as:

E_b_ = E_surf+A_ − E_surf_ − E_A_ (5)

where E_surf+A_, E_surf_, and E_A_ are the total energies of the surface with adsorbate, the total energy of the surface, and the total energy of an isolated adsorbate, respectively.

KMCLib v1.1 [34] was used for all Kinetic Monte Carlo (KMC) simulations. All elementary processes (rare events) with their assumed reaction rates, along with the initial configuration of the system, defined on a regular grid in space, were provided to the code. The systems were then propagated in time on the free energy landscape defined by the provided elementary processes and rates. Simulations were provided enough time to equilibrate, and the statistics were then collected, including integral H_2_ production rates and spatial maps of H_2_ production (normalized from 0 to 1). The initial configurations of the systems were defined on a 130 × 130-points grid which was first populated with metallic sites (Ni), and then circular islands of oxidized Ni phase (OX) were placed on the surface. The set of elementary processes involved reactions (1–3) and considered possible water dissociation at the Ni|OX interface in such a way that H_ads_ is formed on Ni sites, while OH^−^ does not remain adsorbed at OX (much faster removal of OH^−^ compared to other rates in the system). Thus, the reaction is analogous to the Volmer step but only occurs at the Ni|OX interface. Water dissociation at the Ni|OX interface and clean parts of the Ni surface were considered independent and mutually affected indirectly only by the amount of H_ads_ at Ni sites. In addition, surface diffusion of H_ads_ at non-modified parts of the Ni surface was also included in the model. As explained in previous works [15,35], the rates of the considered elementary processes were 10^n^ (with n being an integer number), and were then systematically varied to analyze the effects of the rates of the elementary processes on the total H_2_ production rate. 

## 3. Results

### 3.1. Properties of the Deposited Ni Films

**Figure 1:**
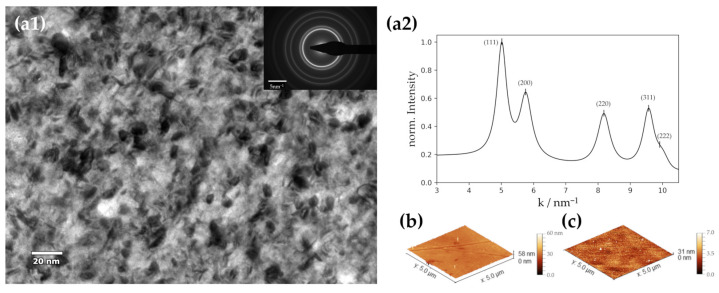
TEM and AFM investigation of a Ni thin film deposited at 80 W. (**a1**) TEM bright-field image and inset: SAED pattern, (**a2**) obtained peaks from SAED, (**b**) AFM image of a 50 nm-thick Ni film, (**c**) AFM image of a 300 nm-thick Ni film.

Ni thin layers were fabricated via magnetron sputter deposition on a conductive and polished Cu substrate. The deposition time was chosen to produce layers of 50 nm and 300 nm, allowing us to analyze the influence of film thickness on the catalytic behavior. Using laser confocal microscopy (LEXT), real obtained thin film thicknesses were confirmed. Subsequent calculations revealed a deposition rate of 11.6 nm min^−1^. Freshly produced films were further characterized via TEM imaging (Figure 1(a1)). The TEM bright-field image shows a contrast sensitive to the grain orientations and therefore reveals that the synthesized Ni thin films are nanocrystalline with an estimated mean grain size of ~9 nm. This small grain size and, therefore, high density of grain boundaries (GBs) has already been found to potentially have a positive influence on the catalytic activity towards HER [36,37]. It was shown in a combined DFT and experimental study by Jiang et al. that the GBs efficiently affect the activity of Au catalysts. As a consequence, it is concluded that a high density of GBs can be traced to an increased ability to adsorb hydrogen and stabilize H* during the reaction steps [37]. The accompanying selected area electron diffraction (SAED) pattern and the corresponding profile obtained by azimuthal integration [38] confirm the polycrystalline fcc structure of the sputter-deposited film (Figure 1a). In addition to examining microstructure and crystal structure, the surface topography was also investigated. AFM of the as-deposited films revealed a homogenous surface coverage of a dense Ni layer on the Cu substrate without evidence of periodic pore structures, which would systematically increase the real surface area. As the produced Ni films were tuned in a way to exclude the roughness factor (RF) effect in the evaluation of Ni activity, sputter deposition and substrate preparation were optimized for obtaining low-roughness samples. The root mean square roughness (RMS) of the Ni films was determined to be around 2 nm using AFM (Figure 1b,c), confirming a successful optimization of the thin film properties. The residual roughness mainly originates from the substrate and its preparation technique rather than the deposition settings. Thus, produced Ni layers exhibit roughness factors (RF) practically equal to 1 (Table 1), allowing a precise determination of kinetic HER parameters without the uncertainties related to electrochemical active surface area determination.

### 3.2. HER Catalysis by the Deposited Ni Films

Following extensive characterization of the initial state, low-roughness Ni thin films were analyzed according to their electrocatalytic behavior towards the HER. First, the HER activity of as-deposited films was determined. Next, the electrode surface was intentionally oxidized by polarizing the working electrode in the anodic regime. This step was performed immediately after the HER analysis without removing the electrodes from the electrolyte between subsequent steps. This in-situ oxidation treatment was followed by another analysis of the HER activity following the same procedure as described for the first step. 

**Figure 2:**
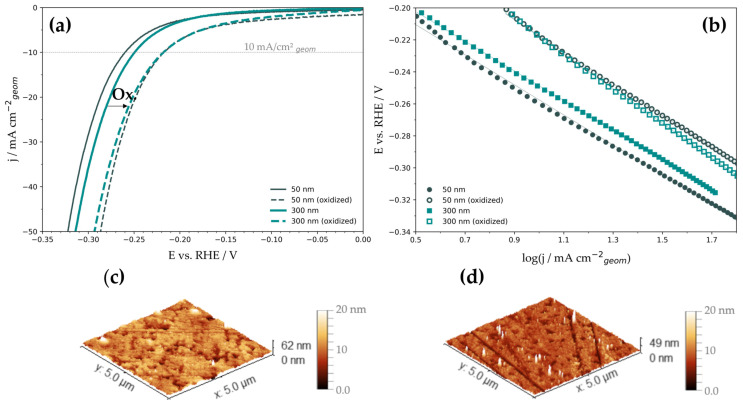
Electrochemical analysis of Ni thin films and topology analysis. (**a**) HER polarization curves before and after oxidation, the arrow indicates the boost of HER upon oxidation. (**b**) Tafel plots (arrows indicate HER activity improvement), (**c**) AFM image of a 50 nm-thick film after cycling to anodic potentials, (**d**) AFM image of a 300 nm-thick film after cycling to anodic potentials.

The reason for the application of this exact method is the anodic oxidation of the pure Ni catalysts, as it has been reported that Ni/Ni(OH)_2_ and Ni/NiO catalysts develop high efficiencies towards the HER [24,39,40,41,42]. The formation of the surface oxides starts with the formation of α-Ni(OH)_2_ at low anodic potentials. This formation is reversible between 0.30–0.50 V vs. RHE, oxidizing and reducing Ni to Ni(II) and forming α-Ni(OH)_2_ and NiO [43,44,45]. In the region of 0.7–1.2 V vs. RHE, α-Ni(OH)_2_ is irreversibly transformed to β-Ni(OH)_2_ [46]. This process is featureless in the CV of the Ni electrode. Moreover β-Ni(OH)_2_ can be derived by direct oxidation of Ni [44]. Alsabet et al. concluded from XPS analysis that the formed oxide layers consist predominantly of β-Ni(OH)_2_ and NiO, while the hydroxide presents the outermost layer, and NiO is considered an interlayer between Ni and β-Ni(OH)_2_. With increasing potential, the oxide layer is prone to growth [44]. Above 1.35 V vs. RHE, the reversible formation and reduction of β-NiOOH take place [47]. All those processes considered, the expected oxygen species residing on the surface after the cyclic oxidation and reduction in the anodic regime are assumed to be β-Ni(OH)_2_ and, possibly, underlying NiO. Both of the named species can potentially influence the catalytic activity of Ni catalysts. The analysis of the as-deposited thin films in the cathodic regime (Figure 2a) revealed the overpotentials at a current density of −10 mA cm^−2^ (*η*_10_) to be −(0.263 ± 0.003) V for the 50 nm Ni thin film. The 300 nm Ni film developed an overpotential of –(0.256 ± 0.005) V, slightly lower than the overpotential of the 50 nm sample but within the experimental uncertainty (Table 1). After anodic oxidation of the Ni films, as expected, a shift of overpotentials was noticed, yielding −(0.220 ± 0.004) V for the 50 nm Ni films and −(0.215 ± 0.006) V for the 300 nm films. Compared with the initial overpotentials, a clear enhancement of the catalytic activity towards the HER can be observed (Figure 2a). 

The obtained HER polarization curves were further processed using Tafel analysis. The linear sections of the Tafel plots (Figure 2b) were extrapolated to *η* = 0 V to extract exchange current densities (*j*_0_). The summarized HER kinetic data (Table 2) indicate a minor effect of surface oxidation on the Tafel slopes, while the exchange current densities are increased by a factor of 3.2 for the 50 nm film and by 4.8 for the 300 nm film. For the HER on Ni metals of various morphologies, there are reports of Volmer–Tafel and Volmer–Heyrovsky reaction mechanisms, as well as mixed forms [48]. However, at higher overpotentials, where Tafel analysis was performed, the Heyrovsky reaction path is considered the rate-determining step in a successive combination of the Volmer and Heyrovsky mechanism. In contrast, the Tafel step is considered negligible in this potential range [49]. AFM analysis (Figure 2c,d and Figure S3) of the as-deposited thin films showed comparable surface roughness and was only slightly affected by surface oxidation. This finding indicates that the RFs (Table 1) hardly changed upon anodic treatment and remained effectively equal to 1. Thus, the calculated exchange current densities are considered to correspond to those evaluated for the electrochemically active surface area. 

Moreover, AFM did not indicate any difference in the porosity comparing dense sputter-deposited Ni films before and after oxidation. We believe that the presented exchange current densities are qualified to be considered reliable values for future reference due to the minimization of uncertainties in real surface estimation by careful experimental design. Interestingly, the obtained *η*_10_ goes along with established data presented by McCrory et al. who set the benchmark for Ni catalysts for the HER to *η*_10_ = −0.26 V, despite featuring a much higher RF [27], as well as with some other high surface area Ni-based cathodes [48,49,50,51]. These findings could indicate that increasing roughness factors can improve the catalytic activity to a certain point, but pushing boundaries for HER activity requires additional strategies to amplify the intrinsic catalytic activity to approach or even outperform noble Pt catalysts. One possibility to do so is surface oxidation, as shown herein. Despite HER activity being significantly improved upon oxidation, it is still quite far from the Pt benchmark (Table 1).

### 3.3. Surface Chemical Composition

**Figure 3:**
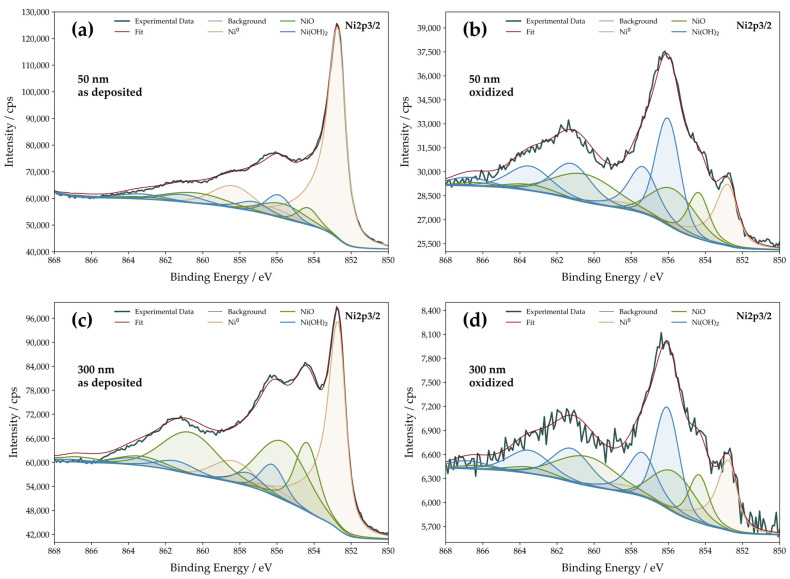
Ni 2p XPS spectra of Ni thin films. The top row (**a**,**b**) gives the spectra of 50 nm-thick films before and after oxidation, while the bottom row (**c**,**d**) gives analogous spectra for 300 nm-thick films. The fitting envelopes of the found species are distinguished by color.

For the boosted catalytic activity, two main surface properties are assumed to have a decisive influence. Both morphology of the surface and the chemical composition do effectively alter the HER mechanism [48,49,50,51]. Primarily, through the oxidation treatment, roughening of the surface could occur and might impact the overall catalytic activity. However, measured RMS and RFs of the samples were found to be practically unaffected by surface oxidation. Thus, increased roughness enhancing the catalytic activity can be evidently excluded, leaving the surface chemical composition the relevant tuning factor to be monitored closely. XPS measurements were conducted to detect the available surface species on the as-deposited Ni thin films and after the in-situ oxidation process, allowing a better understanding of the origin of the increased HER activity (Figure 3).

The evaluation of XPS spectra was done via curve fitting using Ni^0^, NiO, and Ni(OH)_2_ specific fitting envelopes. The sharp peak at a binding energy (BE) of 852.7 eV can be assigned to Ni^0^ in all recorded curves [52,53,54]. For both samples, it decreases strongly in relative height upon oxidation. 

The peaks occurring at BE 854.4 eV and BE 856.2 eV originate from Ni^2+^, and the peak at the lower BE can be assigned to NiO, while the latter partly originates from Ni in Ni(OH)_2_ [44,55]. The satellite peaks at BE 861.4 eV support the assignment and availability of Ni(OH)_2_ on the surface [52,53,54,55], while the satellite at BE 858.4 eV is attributed to Ni^0^ [53]. Table 2 summarizes the Ni surface species found on the studied catalytic films and their relative contributions. 

Analyzing the XPS spectra of the as-deposited Ni thin films reveals that the Ni^0^ peak is the dominant species displayed in the Ni 2p spectra. However, peaks for Ni^2+^ are also detected, indicating the presence of a native oxide layer on the sputter-deposited Ni surface. Upon intended electrochemical oxidation of the surface, a decrease in the relative intensity of Ni^0^ can be monitored. Therefore, Ni(OH)_2_ presents the most prominent species upon oxidation and further thin film electrochemical processing. However, the Ni^0^ peak is still detectable after oxidation of the surface, indicating that the Ni(OH)_2_ and NiO layers might not form a continuous thin film with complete surface coverage. The coexistence of those three species on the catalyst surface seems to contribute simultaneously to lowering the HER overpotential. It is already well established that the presence of elements and structures in addition to pure Ni can lead to a synergistic effect, which can tailor catalytic sites and lead to higher catalytic efficiency [23]. This interplay of Ni and Ni(OH)_2_ has been shown by Danilovic et al., who stated the synergistic effects on bifunctional metal/metal hydroxide surfaces [40]. Also, the less prominent NiO is known to be capable of producing a comparable effect [25,41,42,50,56]. Here, we see (Table 2) that both Ni films have very similar surface composition upon surface oxidation, varying by less than 2 at.%. However, the 300 nm-thick as-deposited films have significantly higher NiO content than the 50 nm-thick counterparts. This finding could explain the slightly higher HER activity of the as-deposited 300 nm Ni films (Figure 2a), which is also visible in the evaluation of survey spectra (Figure S1). The higher NiO content might result from prolonged deposition time, increasing the probability of contamination by oxygen species. However, the pronounced formation of Ni(OH)_2_ in both cases can be associated assuredly with increased HER activity. Additionally, the theory of Ni(OH)_2_ formation is supported by collected O 1 s spectra (Figure S2). The two arising peaks are attributed to NiO (529.6 eV) and NiO in combination with Ni(OH)_2_ (531.6 eV), while the asymmetry of the latter is caused by adsorbents and defective sites [52]. The strong decrease of the pure NiO peak at lower energies and the simultaneous relative growth of the mixed oxide and hydroxide peaks indicate Ni(OH)_2_ formation on the surface.

Although the Ni films were carefully produced to have RF values close to 1 and to eliminate other possible factors that make the understanding of the HER on oxidized Ni surfaces difficult, it is still elusive what the key factors in boosting HER activity are and what is their mutual interplay. Moreover, XPS is a surface-sensitive technique, but it is not limited to the uppermost atomic layer only. It is, thus, challenging to state with certainty the exact fractions of Ni species observed by XPS at the Ni|electrolyte interface.

### 3.4. DFT Modeling and KMC Simulations

Surface oxides and hydroxides can increase the HER reaction rate by boosting the Volmer reaction step via increasing the dissociation of water and adsorption of hydrogen species on the catalyst surface [40,50]. It is believed that in the given surface scenario, water adsorption will occur at Ni species via the interaction of O with Ni oxide species and H with pure Ni on the surface. After the dissociation of the water molecule, Ni facilitates the adsorption and recombination of hydrogen [56]. Moreover, the multifunctional catalyst oxide domains might mediate OH^−^ desorption from the catalyst surface [21,56,57]. 

**Figure 4:**
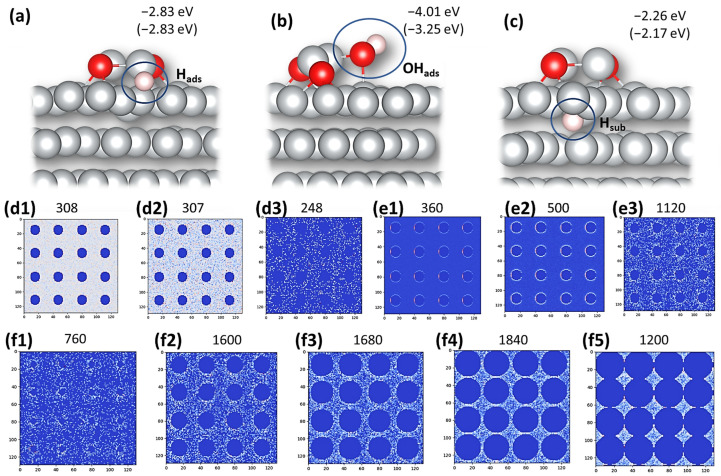
DFT and KMC modeling of an oxide-modified Ni catalyst surface. (**a**) Optimized structure of H_ads_ on (NiO)_2_@Ni(111), (**b**) optimized structure of OH_ads_ on (NiO)_2_@Ni(111), and (**c**) subsurface H (H_sub_) in (NiO)_2_@Ni(111). The indicated numbers give the corresponding binding energies, while values in parentheses give the corresponding Eb for clean Ni(111). (**d1**–**d3**) Spatial maps of H_2_ production when the OX|Ni interface is not active for H_2_O dissociation with increasing H_ads_ diffusion rate, (**e1**–**e3**) spatial maps of H_2_ production when the OX|Ni interface is active for H_2_O dissociation with increasing H_ads_ diffusion rate, (**f1**–**f5**) spatial maps of H_2_ production, when the OX|Ni interface is active for H_2_O dissociation and H_ads_ diffusion is fast with increasing coverage of Ni by the OX phase. The numbers above the maps (**d**–**f**) give integral H_2_ production rates (in a.u.).

To better understand the effect of the Ni surface modification by oxygen-containing species, we performed a series of DFT calculations on Ni(111) and (NiO)_2_@Ni(111) surfaces, comparing how H and OH interact with these surfaces. We found that H_ads_ prefers Ni(111) terraces on both surfaces and that the H_ads_ binding energy is unaffected by the presence of oxide island (NiO)_2_ (Figure 4a). OH_ads_ was found to prefer a bridge position between partially oxidized Ni atoms from (NiO)_2_ islands and Ni atoms from the Ni(111) surface (Figure 4b). The interaction of OH_ads_ with the (NiO)_2_-modified surface is significantly stronger compared with clean Ni(111) (−4.01 eV vs. −3.25 eV). The linearity of the relation between the activation energy barrier for water dissociation and the enthalpy change of water dissociation [58] leads to an acceleration of the Volmer step (1) on an oxide-modified surface compared to clean Ni(111) surfaces. Importantly, our findings suggest that the effect is solely due to the enhanced interaction of OH_ads_ with the oxidized phase, while the impact of H_ads_ energetics is negligible. Under electrochemical conditions, OH^−^ is formed upon dissociation (1). It is unstable at the negative potentials applied for the HER and is, therefore, desorbed off the surface. Considering the formation of the subsurface hydride on clean and modified Ni(111), we found that H_sub_ (Figure 4c) is energetically more favorable for a modified surface.

Moreover, the literature suggests that the availability of surface oxides mediates the amount of H_ads_ and OH_ads_ and promotes H_ads_ recombination via the Tafel reaction (3) [21,56]. To investigate this possibility, we performed a set of KMC simulations for the Volmer–Tafel mechanism of H_2_ evolution (Figure 4d–f). In the first scenario, the surface modifier (OX phase) does not contribute to the water dissociation. When the rate of H_ads_ surface diffusion increases, the total H_2_ production rate decreases (Figure 4(d1–d3)). This is due to the high mobility of H_ads_, reducing the probability of H_ads_ recombination. In the second set, the surface modifier (OX phase) allows for fast H_2_O dissociation at the OX|Ni interface. Increasing H_ads_ mobility in this scenario boosts the total H_2_ production (Figure 4(e1–e3)), as H_ads_ is allowed to diffuse away from its formation site. Empty OX|Ni interfacial sites allow the creation of more H_ads_ and increase H_ads_ concentration on the surface. This raises the probability of the H_ads_ recombination tremendously. In this case, H_2_ is not only produced at the OX|Ni interface but all over the Ni surface (Figure 4(e2) vs. Figure 4(e3)). It can, therefore, be concluded that if the presence of a surface modifier increases the mobility of H_ads_, both the H_2_O dissociation rate as well as the H_2_ production will increase significantly. For comparison (Figure 4(di) vs. Figure 4(ei) (i = 1, 2, or 3)), the rates of all elementary processes were chosen equally (including H_ads_ diffusion rates), except for the H_2_O dissociation at the OX|Ni interface, which was assumed 10,000 times faster in the second scenario. Dissociation acceleration gives H_2_ production enhancement factors close to those experimentally observed by comparing *j*_0_ before and after oxidation of the Ni films. In a third scenario, we investigated how the surface coverage by a modifier (OX phase) affects the H_2_ production rate. We note that in our simulations, H_2_ cannot be produced at the OX phase surface. Therefore, the modifier’s only contribution is the mediation of H_2_O dissociation at the OX|Ni interface. In this set of simulations, H_ads_ diffusion was considered as fast as in previous simulations. It was found that an increase in the OX surface fraction leads to an increase in the H_2_ production rate. This accelerating effect passes through a maximum governed by Ni surface coverage, unfolding in the finding that an optimal OX phase coverage provides a maximum H_2_ production rate. The existence of this maximum is the result of an interplay between the length of the OX|M interface at which H_2_O dissociates and the number of Ni sites at which H_ads_ is formed and can recombine to H_2_. In practice, this means that the concentration of oxidized Ni phase on Ni catalysts can/should be carefully optimized to provide a maximum enhancement of HER activity. 

### 3.5. HER Activity upon Prolonged Cycling—The Crucial Role of Surface Oxidation

**Figure 5:**
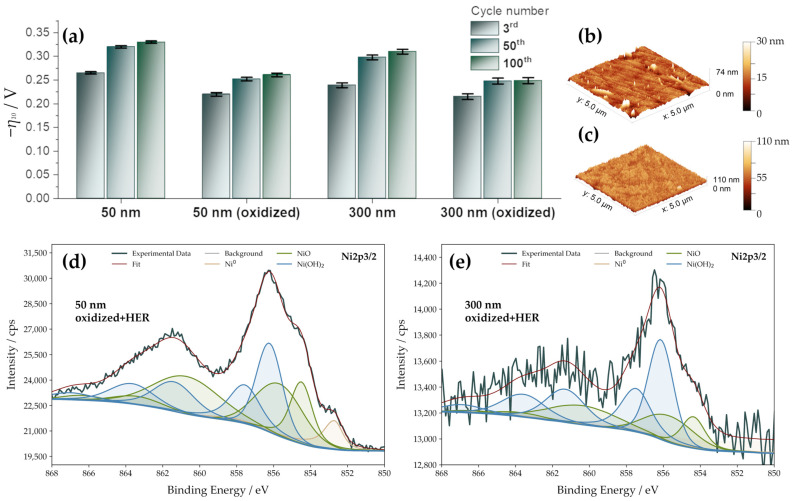
Catalyst durability and topology evolution. (**a**) HER overpotentials (*η*_10_) for as-deposited and oxidized films (3rd, 50th, and 100th cycle), (**b**) AFM image of oxidized 50 nm-thick films after 100 cycles (5 μm × 5 μm spot), (**c**) AFM image of oxidized 300 nm-thick films after 100 cycles (5 μm × 5 μm spot), (**d**,**e**) Ni 2p XPS of 50 nm and 300 nm-thick Ni films after oxidation and three cycles of hydrogen evolution. The fitting envelopes of the found species are distinguished by color.

In addition to the initial activity of a catalyst, its stability is also a crucial factor to be closely monitored. The stability of the Ni thin films was tested via CV in the cathodic regime. The electrodes were cycled to deep negative potentials, typically reaching ~−50 mA cm^−2^. The electrodes were switched 100 times between intense HER and potentials corresponding to the formation of α-Ni(OH)_2_ and NiO. Considering the potential scan rate, each experiment took about 8 h. The results (Figure 5a) show a continuous overpotential increase upon cycling. However, the observed activity loss is more pronounced for as-deposited films in comparison to the oxidized films. Even after 50 cycles, the overpotentials of oxidized samples still outperform the corresponding as-deposited films (Figure 5a).

AFM analysis shows more prominent topography changes after 100 cycles and confirms an increasing RMS roughness to 4.1 nm (50 nm films) and 12 nm for 300 nm films (Figure 5b,c). Surface roughening occurs after the oxidation step and after 100 cycles, each reaching intense H_2_ evolution. Still, the roughness factors remain rather low. For 50 nm films, RF is found to be 1.2, and for 300 nm films, RF is 1.6. Higher RFs for 300 nm Ni films align with higher HER activities. The surface oxidation associated with stable Ni(OH)_2_ formation (Table 2) is crucial to maintain HER activity. We have not observed any substantial changes in the surface topology. However, we note that samples were not exposed to very high currents (up to −50 mA cm^−2^) during the testing. The possibility of more pronounced structural changes at industry-relevant currents cannot be excluded at this point and is beyond the scope of the present work. Despite the overall activity decrease trend during cycling, the oxidized thin films preserve superior activity compared to their as-deposited counterparts. For as-deposited films, after 100 cycles, *η*_10_ is shifted by −65 mV and −71 mV for 50 nm and 300 nm-thick films. For oxidized films, *η*_10_ shifts less, specifically by −41 mV (50 nm films) and −34 mV (300 nm films). Moreover, the state of the Ni surface evolves under HER conditions (Table 3), surprisingly increasing the total NiO and Ni(OH)_2_ content at the expense of Ni^0^. The evolution of the chemical composition depends on the film thickness, even though the surface chemical composition of both 50 nm and 300 nm films was very similar after the oxidation (Table 2).

**Figure 6:**
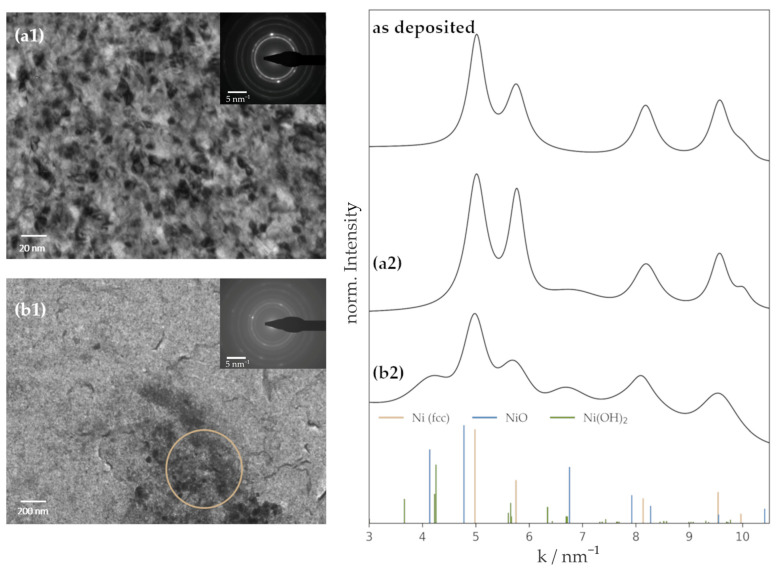
TEM and SAED analysis of a 50 nm Ni film after 100 cycles of the stability test. (**a1**) TEM bright-field image of a representative spot and (**b1**) of surface alterations (highlighted region) as well as corresponding diffraction profiles obtained by azimuthal integration of the SAED patterns (**a2**,**b2**). The profile of the as-deposited layer and theoretical reference peaks are displayed for comparison [62].

We believe that cycling leads to mechanical stresses within the Ni layer upon oscillating lattice expansion and subsequent contraction, which is most likely responsible for surface roughening. This emphasizes the possibility of either hydrogen incorporation into interstitial sites or even the formation of a NiH phase in the catalyst. The hydrogen uptake could, therefore, lead to oscillating lattice deformation and potentially causes damage in the sputtered layers promoting delamination. That mode of catalyst deactivation via NiH formation has already been described in several studies and can be attributed to a change in electronic surface structure [59,60]. Thus, the activity loss can be linked to the uptake and incorporation of hydrogen into Ni, forming hydrides on the metal surface and within the thin film [45,61], despite the fact that no direct observation of hydrides was possible in this work. Influencing the density of states on the surface, NiH is considered to increase the overpotentials and form a barrier for hydrogen diffusion within the catalyst [45,59,60,61]. However, we note that the DFT calculations suggest that subsurface hydride is more stable in modified Ni(111) (Figure 4c) compared to clean Ni(111) by 0.09 eV, but this result relates to thermodynamic stability only. Thus, the question remains whether the oxidized Ni phases might induce an additional barrier for H_ads_ diffusion to subsurface sites, which could explain slower activity loss for the oxidized films. Using TEM and SAED analysis, no residual NiH after the cycling stability tests was observed. On the other hand, structural inhomogeneities arise in the films, which present themselves as NiO and Ni(OH)_2_-rich islands on the surface. This inhomogeneous coverage with oxide and hydroxide species supports the XPS results, indicating the coexistence of Ni^0^ and its oxide species on the surface. The detection of in-situ-formed NiH might be challenging, especially considering the presence of oxides and hydroxides (Figure 6). This issue is yet to be addressed, and it is particularly challenging to resolve it as the as-deposited Ni films are also clearly partially oxidized (Table 2).

At the same time, the surface phases present under electrochemical conditions could be different from those observed in ex-situ experiments such as XPS or SAED.

## 4. Conclusions

Ni thin films of different thicknesses were prepared via magnetron sputter deposition to minimize roughness and properly evaluate the intrinsic catalytic activity of Ni towards HER in alkaline solutions. TEM investigations revealed a Ni fcc nanocrystalline structure of the films with a grain size of about 9 nm. AFM images confirmed the deposition of smooth films with extremely low roughness factors close to 1. HER activities of 50 nm and 300 nm-thick Ni films are comparable. To study the premise of enhanced HER due to the presence of oxide phases, the surface chemical composition was tuned via electrochemical oxidation performed by cyclic voltammetry in the anodic regime to study the premise of enhanced HER due to the presence of oxide phases. XPS studies confirmed a change of the exposed surface species and, therefore, a successful alteration of Ni surface-active species upon oxidation, resulting in a high surface content of Ni(OH)_2_, irrespective of film thickness. On the other hand, negligible surface roughening is seen, allowing for precise evaluation of exchange current densities for as-deposited and oxidized films. The applied oxidation process shifts the onset of hydrogen evolution towards lower overpotentials and increases the exchange current densities 3–5 times. DFT calculations correlated the boost of HER activity with enhanced OH adsorption at the oxide|nickel interface, while no impact of oxide species on the H_ads_ energetics was observed. Additionally, KMC simulations proved that speeding up H_2_O dissociation at the oxide|nickel interface enhances the H_2_ production rate, even if the rates of other processes in the overall mechanism remain unchanged. The H_ads_ surface diffusion rate on the nickel surface also plays a significant role. The maximum H_2_ production rate can be reached when the number of oxide|nickel sites for H_2_O dissociation is appropriately balanced with the H_ads_ surface diffusion rate. The results imply that surface oxidation is crucial for retaining high HER activity under a prolonged operation of a nickel catalyst. During this process, the surface roughness increases while structural inhomogeneities arise in the deposited Ni films, which can be associated with Ni hydride formation under HER conditions. The presented results contribute to a better understanding of the HER on nickel in alkaline media and give additional guidelines for boosting the HER performance of Ni-based cathodes.

## Figures and Tables

**Table 1:** Ni thin film electrochemical data for 50 and 300 nm films in alkaline solution (1 M KOH). Overpotentials at −10 mA cm^−2^ *η*_10_ were evaluated and compared to benchmark catalysts. Tafel slopes (*b*) and exchange current densities (*j*_0_) were extracted using Tafel analysis. Roughness factors were obtained using AFM analysis as the ratio between the real surface obtained by AFM and the geometric surface area of scanned spots. In ref. [27], RFs were obtained using the double-layer charging method.

Film Thickness	Film State	*η*_10_ (V)	*b* (mV dec^−1^)	log(*j*_0_/A cm^−2^_geom_)	RF
50 nm	as-deposited	−(0.263 ± 0.003)	−(89 ± 4)	−(4.92 ± 0.15)	1.004 ± 0.071
	oxidized	−(0.220 ± 0.004)	−(92 ± 2)	−(4.42 ± 0.13)	1.006 ± 0.070
300 nm	as-deposited	−(0.256 ± 0.005)	−(92 ± 3)	−(4.71 ± 0.14)	1.004 ± 0.070
	oxidized	−(0.215 ± 0.006)	−(108 ± 5)	−(4.03 ± 0.12)	1.011 ± 0.071
Benchmarks [27]					
Ni (electrodeposited)		−(0.26 ± 0.04)	/	/	16 ± 2
Pt (electrodeposited)		−(0.10 ± 0.02)	/	/	10 ± 2

**Table 2:** Relative contributions (in at.%) of different Ni species identified by XPS.

Film Thickness	Film State	Ni^0^	Ni^2+^ [NiO]	Ni^2+^ [Ni(OH)_2_]
50 nm	as-deposited	65.6	18.2	16.2
	oxidized	15.4	34.5	50.1
300 nm	as-deposited	39.2	47.9	12.9
	oxidized	16.4	35.3	48.3

**Table 3:** Relative contributions (in at.%) of different Ni species identified by XPS of oxidized thin films after three cycles of hydrogen evolution reaction (Figure 5d,e).

Film Thickness	Film State	Ni^0^ [at.%]	Ni^2+^ (NiO) [at.%]	Ni^2+^ (Ni(OH)_2_) [at.%]
50 nm	oxidized + HER	6.6	48.9	44.5
300 nm	oxidized + HER	4.9	33.4	61.7

## Data Availability

The data presented in this study are available on request from the corresponding author.

## Supplementary Materials

The following supporting information can be downloaded at: https://www.mdpi.com/article/10.3390/nano13142085/s1, Figure S1: XPS survey spectra of Ni thin films; Figure S2: O 1 s XPS of Ni thin films; Figure S3: AFM topography images of Ni thin films.

## References

[1] Dawood F., Anda M., Shafiullah G.M. (2020). Hydrogen Production for Energy: An Overview. Int. J. Hydrogen Energy.

[2] IEA (2021). Global Hydrogen Review 2021.

[3] Suen N.-T., Hung S.-F., Quan Q., Zhang N., Xu Y.-J., Chen H.M. (2017). Electrocatalysis for the oxygen evolution reaction: Recent development and future perspectives. Chem. Soc. Rev..

[4] Wang S., Lu A., Zhong C.-J. (2021). Hydrogen production from water electrolysis: Role of catalysts. Nano Converg..

[5] Greeley J., Jaramillo T.F., Bonde J., Chorkendorff I., Nørskov J.K. (2006). Computational high-throughput screening of electrocatalytic materials for hydrogen evolution. Nat. Mater..

[6] Sabatier P. (1911). Hydrogénations et déshydrogénations par catalyse. Eur. J. Inorg. Chem..

[7] Gebremariam G.K., Jovanović A.Z., Dobrota A.S., Skorodumova N.V., Pašti I.A. (2022). Hydrogen Evolution Volcano(es)—From Acidic to Neutral and Alkaline Solutions. Catalysts.

[8] Lasia A. (2003). Hydrogen Evolution Reaction. Handbook of Fuel Cells, Fundamentals, Technology and Applications.

[9] Ferriday T.B., Middleton P.H., Kolhe M.L. (2021). Review of the Hydrogen Evolution Reaction—A Basic Approach. Energies.

[10] Shen L.-F., Lu B.-A., Qu X.-M., Ye J.-Y., Zhang J.-M., Yin S.-H., Wu Q.-H., Wang R.-X., Shen S.-Y., Sheng T. (2019). Does the oxophilic effect serve the same role for hydrogen evolution/oxidation reaction in alkaline media?. Nano Energy.

[11] Strmcnik D., Uchimura M., Wang C., Subbaraman R., Danilovic N., van der Vliet D., Paulikas A.P., Stamenkovic V.R., Markovic N.M. (2013). Improving the hydrogen oxidation reaction rate by promotion of hydroxyl adsorption. Nat. Chem..

[12] Jiao Y., Zheng Y., Jaroniec M., Qiao S.Z. (2015). Design of electrocatalysts for oxygen- and hydrogen-involving energy conversion reactions. Chem. Soc. Rev..

[13] Mahmood N., Yao Y., Zhang J.-W., Pan L., Zhang X., Zou J.-J. (2018). Electrocatalysts for Hydrogen Evolution in Alkaline Electrolytes: Mechanisms, Challenges, and Prospective Solutions. Adv. Sci..

[14] Wang X., Zheng Y., Sheng W., Xu Z.J., Jaroniec M., Qiao S.-Z. (2020). Strategies for design of electrocatalysts for hydrogen evolution under alkaline conditions. Mater. Today.

[15] Gutić S.J., Metarapi D., Jovanović A.Z., Gebremariam G.K., Dobrota A.S., Vasiljević B.N., Pašti I.A. (2023). Redrawing HER Volcano with Interfacial Processes—The Role of Hydrogen Spillover in Boosting H_2_ Evolution in Alkaline Media. Catalysts.

[16] Coutanceau C., Baranton S., Audichon T. (2018). Hydrogen Production from Water Electrolysis. Hydrogen Energy and Fuel Cells Primers, Hydrogen Electrochemical Production.

[17] David M., Ocampo-Martínez C., Sanchez-Pena R. (2019). Advances in alkaline water electrolyzers: A review. J. Energy Storage.

[18] Zeng K., Zhang D. (2010). Recent progress in alkaline water electrolysis for hydrogen production and applications. Prog. Energy Combust. Sci..

[19] Oshchepkov A.G., Bonnefont A., Savinova E.R. (2021). Metal–metal (hydr)oxide heterostructures for electrocatalysis of hydrogen electrode reactions. Curr. Opin. Electrochem..

[20] Zhu Y., Lin Q., Zhong Y., Tahini H.A., Shao Z., Wang H. (2020). Metal oxide-based materials as an emerging family of hydrogen evolution electrocatalysts. Energy Environ. Sci..

[21] Subbaraman R., Tripkovic D., Chang K.-C., Strmcnik D., Paulikas A.P., Hirunsit P., Chan M., Greeley J., Stamenkovic V., Markovic N.M. (2012). Trends in activity for the water electrolyser reactions on 3d M(Ni,Co,Fe,Mn) hydr(oxy)oxide catalysts. Nat. Mater..

[22] Xu H., Li F., Li Z., Liu Q., Li B., Xu L., He G. (2022). Research Progress of Metal Oxide as Cathode Materials for Hydrogen Evolution. Int. J. Electrochem. Sci..

[23] Gong M., Wang D.-Y., Chen C.-C., Hwang B.-J., Dai H. (2016). A mini review on nickel-based electrocatalysts for alkaline hydrogen evolution reaction. Nano Res..

[24] Gong M., Zhou W., Tsai M.-C., Zhou J., Guan M., Lin M.-C., Zhang B., Hu Y., Wang D.-Y., Yang J. (2014). Nanoscale nickel oxide/nickel heterostructures for active hydrogen evolution electrocatalysis. Nat. Commun..

[25] Xie K., Guo P., Xiong Z., Sun S., Wang H., Gao Y. (2021). Ni/NiO hybrid nanostructure supported on biomass carbon for visible-light photocatalytic hydrogen evolution. J. Mater. Sci..

[26] Nečas D., Klapetek P. (2012). Gwyddion: An open-source software for SPM data analysis. Open Phys..

[27] McCrory C.C.L., Jung S., Ferrer I.M., Chatman S.M., Peters J.C., Jaramillo T.F. (2015). Benchmarking Hydrogen Evolving Reaction and Oxygen Evolving Reaction Electrocatalysts for Solar Water Splitting Devices. J. Am. Chem. Soc..

[28] Kresse G., Hafner J. (1993). *Ab initio* molecular dynamics for liquid metals. Phys. Rev. B.

[29] Kresse G., Furthmüller J. (1996). Efficiency of ab-initio total energy calculations for metals and semiconductors using a plane-wave basis set. Comput. Mater. Sci..

[30] Kresse G., Furthmüller J. (1996). Efficient iterative schemes for ab initio total-energy calculations using a plane-wave basis set. Phys. Rev. B.

[31] Perdew J.P., Burke K., Ernzerhof M. (1996). Generalized gradient approximation made simple. Phys. Rev. Lett..

[32] Blöchl P.E. (1994). Projector augmented-wave method. Phys. Rev. B.

[33] Monkhorst H.J., Pack J.D. (1976). Special points for Brillouin-zone integrations. Phys. Rev. B.

[34] Leetmaa M., Skorodumova N.V. (2015). KMCLib 1.1: Extended random number support and technical updates to the KMCLib general framework for kinetic Monte-Carlo simulations. Comput. Phys. Commun..

[35] Pašti I.A., Leetmaa M., Skorodumova N.V. (2016). General principles for designing supported catalysts for hydrogen evolution reaction based on conceptual Kinetic Monte Carlo modeling. Int. J. Hydrogen Energy.

[36] Landau M.V., Vidruk R., Vingurt D., Fuks D., Herskowitz M. (2014). Grain boundaries in nanocrystalline catalytic materials as a source of surface chemical functionality. Rev. Chem. Eng..

[37] Jiang R., Fu J., Wang Z., Dong C. (2022). Grain Boundary—A Route to Enhance Electrocatalytic Activity for Hydrogen Evolution Reaction. Appl. Sci..

[38] Gammer C., Mangler C., Rentenberger C., Karnthaler H. (2010). Quantitative local profile analysis of nanomaterials by electron diffraction. Scr. Mater..

[39] Guo W., Kim J., Kim H., Hong S., Kim S., Ahn S. (2022). Electrochemically activated Ni@Ni(OH)_2_ heterostructure as efficient hydrogen evolution reaction electrocatalyst for anion exchange membrane water electrolysis. Mater. Today Chem..

[40] Danilovic N., Subbaraman R., Strmcnik D., Chang K.-C., Paulikas A.P., Stamenkovic V.R., Markovic N.M. (2012). Enhancing the Alkaline Hydrogen Evolution Reaction Activity through the Bifunctionality of Ni(OH)_2_/Metal Catalysts. Angew. Chem. Int. Ed..

[41] Lim D., Kim S., Kim N., Oh E., Shim S.E., Baeck S.-H. (2020). Strongly Coupled Ni/Ni(OH)_2_ Hybrid Nanocomposites as Highly Active Bifunctional Electrocatalysts for Overall Water Splitting. ACS Sustain. Chem. Eng..

[42] Faid A.Y., Barnett A.O., Seland F., Sunde S. (2020). Ni/NiO nanosheets for alkaline hydrogen evolution reaction: In situ electrochemical-Raman study. Electrochim. Acta.

[43] Alsabet M., Grden M., Jerkiewicz G. (2011). Electrochemical Growth of Surface Oxides on Nickel. Part 1: Formation of α-Ni(OH)_2_ in Relation to the Polarization Potential, Polarization Time, and Temperature. Electrocatalysis.

[44] Alsabet M., Grden M., Jerkiewicz G. (2014). Electrochemical Growth of Surface Oxides on Nickel. Part 2: Formation of β-Ni(OH)_2_ and NiO in Relation to the Polarization Potential, Polarization Time, and Temperature. Electrocatalysis.

[45] Hall D.S., Bock C., MacDougall B.R. (2013). The Electrochemistry of Metallic Nickel: Oxides, Hydroxides, Hydrides and Alkaline Hydrogen Evolution. J. Electrochem. Soc..

[46] Grden M., Klimek K., Czerwinski A. (2004). A quartz crystal microbalance study on a metallic nickel electrode. J. Solid State Electrochem..

[47] Alsabet M., Grdeń M., Jerkiewicz G. (2015). Electrochemical Growth of Surface Oxides on Nickel. Part 3: Formation of β-NiOOH in Relation to the Polarization Potential, Polarization Time, and Temperature. Electrocatalysis.

[48] Grdeń M., Jerkiewicz G. (2019). Influence of Surface Treatment on the Kinetics of the Hydrogen Evolution Reaction on Bulk and Porous Nickel Materials. Electrocatalysis.

[49] Krstajić N., Popović M., Grgur B., Vojnović M., Šepa D. (2001). On the kinetics of the hydrogen evolution reaction on nickel in alkaline solution: Part I. The mechanism. J. Electroanal. Chem..

[50] Liang Z., Ahn H.S., Bard A.J. (2017). A Study of the Mechanism of the Hydrogen Evolution Reaction on Nickel by Surface Interrogation Scanning Electrochemical Microscopy. J. Am. Chem. Soc..

[51] Jovanović A.Z., Bijelić L., Dobrota A.S., Skorodumova N.V., Mentus S.V., Pašti I.A. (2022). Enhancement of hydrogen evolution reaction kinetics in alkaline media by fast galvanic displacement of nickel with rhodium—From smooth surfaces to electrodeposited nickel foams. Electrochim. Acta.

[52] Biesinger M.C., Payne B.P., Lau L.W.M., Gerson A., Smart R.S.C. (2009). X-ray photoelectron spectroscopic chemical state quantification of mixed nickel metal, oxide and hydroxide systems. Surf. Interface Anal..

[53] Grosvenor A.P., Biesinger M.C., Smart R.S.C., McIntyre N.S. (2006). New interpretations of XPS spectra of nickel metal and oxides. Surf. Sci..

[54] Biesinger M.C., Payne B.P., Grosvenor A.P., Lau L.W.M., Gerson A.R., Smart R.S.C. (2011). Resolving surface chemical states in XPS analysis of first row transition metals, oxides and hydroxides: Cr, Mn, Fe, Co and Ni. Appl. Surf. Sci..

[55] Nesbitt H.W., Legrand D., Bancroft G.M. (2000). Interpretation of Ni2p XPS spectra of Ni conductors and Ni insulators. Phys. Chem. Miner..

[56] Liu X., Wang X., Yuan X., Dong W., Huang F. (2016). Rational composition and structural design of in situ grown nickel-based electrocatalysts for efficient water electrolysis. J. Mater. Chem. A.

[57] Subbaraman R., Tripkovic D., Strmcnik D., Chang K.-C., Uchimura M., Paulikas A.P., Stamenkovic V., Markovic N.M. (2011). Enhancing Hydrogen Evolution Activity in Water Splitting by Tailoring Li^+^-Ni(OH)_2_-Pt Interfaces. Science.

[58] Michaelides A., Liu Z.-P., Zhang C.J., Alavi A., King D.A., Hu P. (2003). Identification of General Linear Relationships between Activation Energies and Enthalpy Changes for Dissociation Reactions at Surfaces. J. Am. Chem. Soc..

[59] Abouatallah R., Kirk D., Graydon J. (2002). Long-term electrolytic hydrogen permeation in nickel and the effect of vanadium species addition. Electrochim. Acta.

[60] Rommal H.E.G., Morgan P.J. (1988). The Role of Absorbed Hydrogen on the Voltage-Time Behavior of Nickel Cathodes in Hydrogen Evolution. J. Electrochem. Soc..

[61] Soares D.M., Teschke O., Torriani I. (1992). Hydride Effect on the Kinetics of the Hydrogen Evolution Reaction on Nickel Cathodes in Alkaline Media. J. Electrochem. Soc..

[62] Jain A., Ong S.P., Hautier G., Chen W., Richards W.D., Dacek S., Cholia S., Gunter D., Skinner D., Ceder G. (2013). Commentary: The Materials Project: A materials genome approach to accelerating materials innovation. APL Mater..

