# Supplementary material for: Hydrogen Evolution Reaction on Ultra-Smooth Sputtered Nanocrystalline Ni Thin Films in Alkaline Media—From Intrinsic Activity to the Effects of Surface Oxidation
Source: Nanomaterials (Basel). 2023 Jul 17;13(14):2085. doi: 10.3390/nano13142085 (PMC10383487; doi:10.3390/nano13142085)
Supplement: Supplementary file 1 [file nanomaterials-13-02085-s001.zip › nanomaterials-2490792-supplementary.pdf]

Article

Hydrogen Evolution Reaction on Ultra-Smooth Sputtered Nanocrystalline Ni Thin Films in Alkaline Media—From Intrinsic Activity to the Effects of Surface Oxidation

Daniela Neumüller [1], Lidija D. Rafailović [1,*], Aleksandar Z. Jovanović [2], Natalia V. Skorodumova [3,4], Igor A. Pašti [2], Alice Lassnig [5], Thomas Griesser [6], Christoph Gammer [5] and Jürgen Eckert [1,5]

[1] Department of Materials Science, Montanuniversität Leoben, 8700 Leoben, Austria; daniela.neumueller@unileoben.ac.at (D.N.); juergen.eckert@unileoben.ac.at (J.E.)
[2] University of Belgrade-Faculty of Physical Chemistry, 11158 Belgrade, Serbia; a.jovanovic@ffh.bg.ac.rs (A.Z.J.); igor@ffh.bg.ac.rs (I.A.P.)
[3] Department of Materials Science and Engineering, School of Industrial Engineering and Management, KTH–Royal Institute of Technology, 100 44 Stockholm, Sweden; snv123@kth.se
[4] Applied Physics, Division of Materials Science, Department of Engineering Sciences and Mathematics, Luleå University of Technology, 971 87 Luleå, Sweden
[5] Erich Schmid Institute of Materials Science, Austrian Academy of Sciences, 8700 Leoben, Austria; alice.lassnig@oeaw.ac.at (A.L.); christoph.gammer@oeaw.ac.at (C.G.)
[6] Institute of Chemistry of Polymeric Materials, Department of Polymer Engineering and Science, Montanuniversität Leoben, 8700 Leoben, Austria; thomas.griesser@unileoben.ac.at
* Correspondence: lidija.rafailovic@unileoben.ac.at

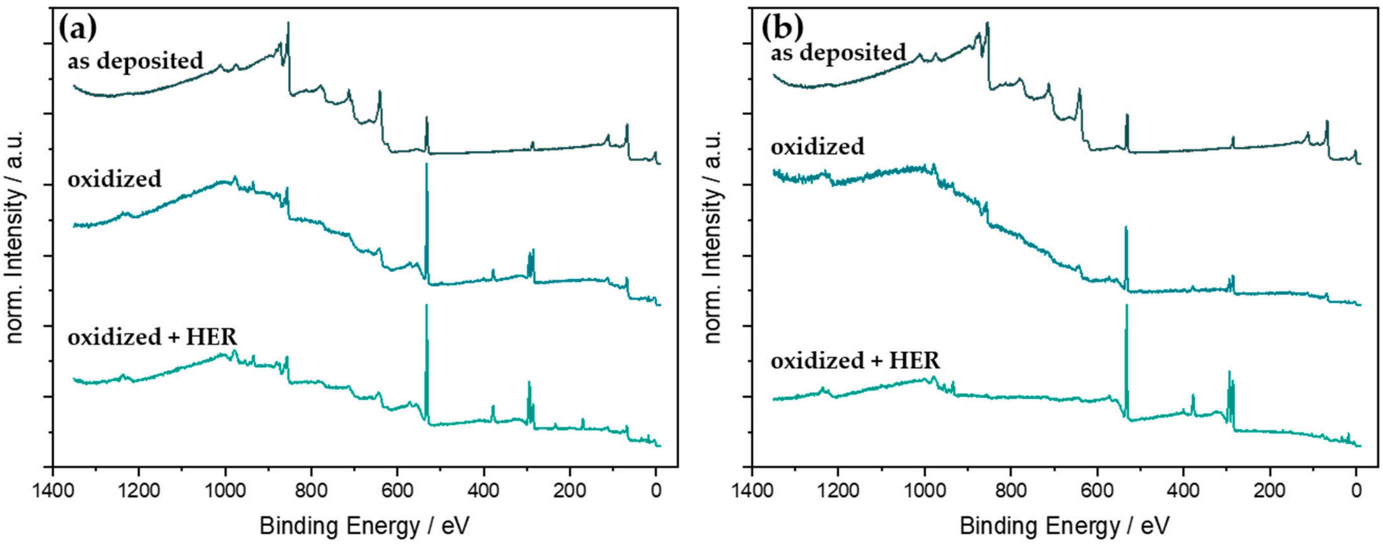

Figure S1. XPS survey spectra of Ni thin films of 50 nm (a) and 300 nm (b). The top row displays the spectra of the as-deposited thin films, followed by the presentation of the XPS survey after oxidation and after oxidation followed by three cycles of HER. Please note that the presented data is collected from separate samples.

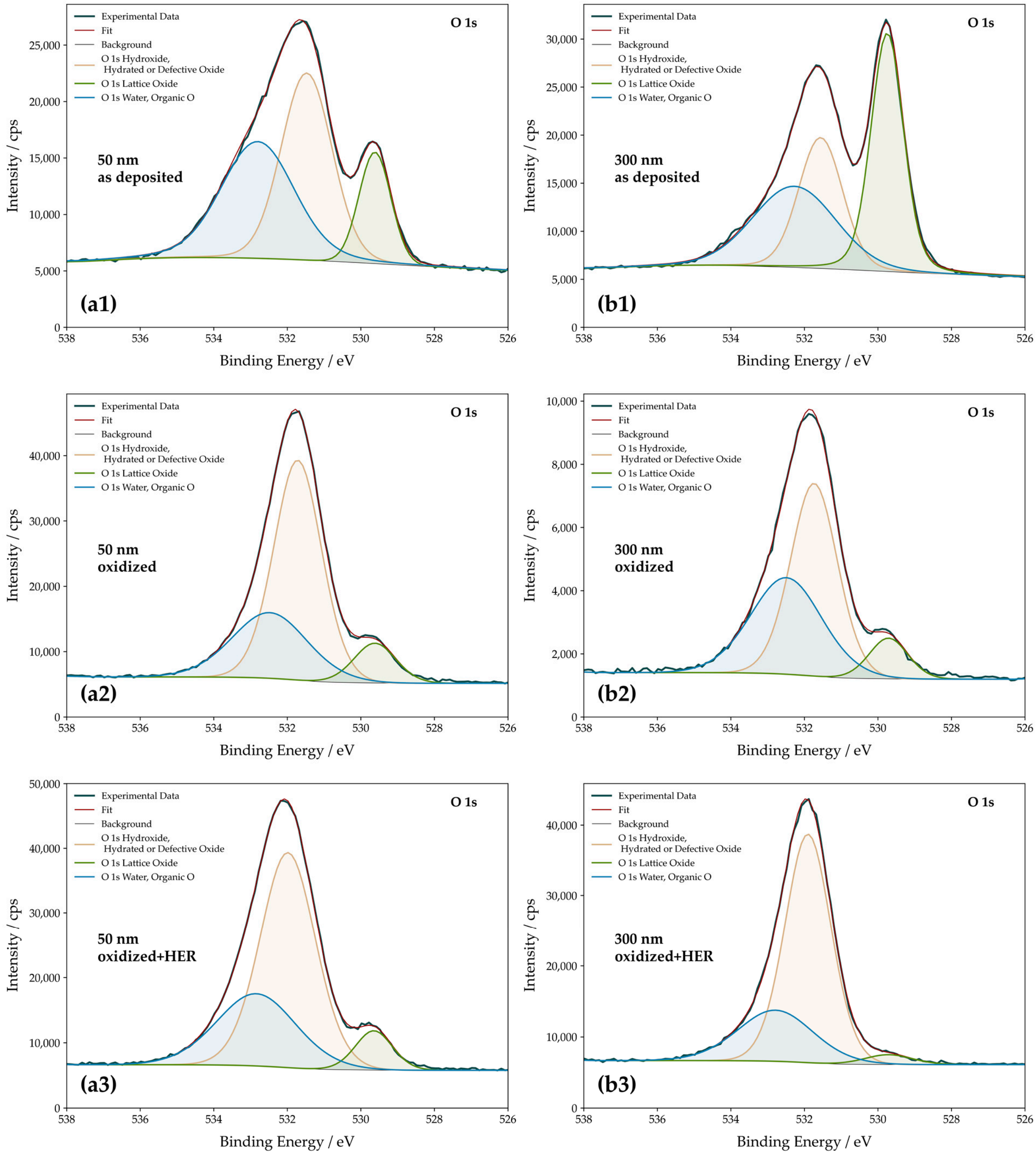

Figure S2. O 1s XPS of Ni thin films of 50 nm (a) and 300 nm (b). The top row displays the spectra of the as-deposited thin films (a1, b1), followed by the presentation of the XPS after oxidation (a2, b2) and after oxidation and three cycles of HER (a3, b3). Please note that the presented data is collected from separate samples.

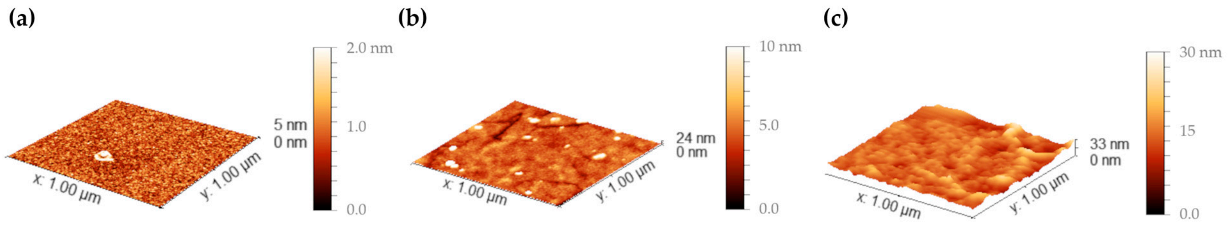

Figure S3. AFM topography images of Ni thin films (a) as-deposited on Si substrate, (b) as-deposited on Cu substrate, (c) after electrochemical oxidation followed by three HER cycles.
